# Genetic and epigenetic silencing of the *beclin 1 *gene in sporadic breast tumors

**DOI:** 10.1186/1471-2407-10-98

**Published:** 2010-03-16

**Authors:** Zidong Li, Bo Chen, Yiqing Wu, Feng Jin, Yongjing Xia, Xiangjun Liu

**Affiliations:** 1 Institute of Biomedical Informatics, School of Medicine, Tsinghua University, Beijing, PR China; 2 Department of Biological Science and Biotechnology, Tsinghua University, Beijing, PR China; 3 Ministry of Education Key Laboratory of Bioinformatics, Tsinghua University, Beijing, PR China; 4 Department of Surgical Oncology, The First Affiliated Hospital, China Medical University, Shenyang, PR China

## Abstract

**Background:**

Beclin 1, an important autophagy-related protein in human cells, is involved in cell death and cell survival. *Beclin 1 *mapped to human chromosome 17q21. It is widely expressed in normal mammary epithelial cells. Although down-regulated expression with mono-allelic deletions of *beclin 1 *gene was frequently observed in breast tumors, whether there was other regulatory mechanism of *beclin 1 *was to be investigated. We studied the expression of beclin 1 and explored the possible regulatory mechanisms on its expression in breast tumors.

**Methods:**

20 pairs of tumors and adjacent normal tissues from patients with sporadic breast invasive ductal cancer (IDCs) were collected. The mRNA expression of *beclin 1 *was detected by real-time quantitative RT-PCR. Loss of heterozygosity (LOH) was determined by real-time quantitative PCR and microsatellite methods. The protein expression of beclin 1, p53, BRCA1 and BRCA2 was assessed by immunohistochemistry. CpG islands in 5' genomic region of beclin 1 gene were identified using MethylPrimer Program. Sodium bisulfite sequencing was used in examining the methylation status of each CpG island.

**Results:**

Decreased *beclin 1 *mRNA expression was detected in 70% of the breast tumors, and the protein levels were co-related to the mRNA levels. Expression of *beclin 1 *mRNA was demonstrated to be much higher in the BRCA1 positive tumors than that in the BRCA1 negative ones. Loss of heterozygosity was detected in more than 45% of the breast tumors, and a dense cluster of CpG islands was found from the 5' end to the intron 2 of the *beclin 1 *gene. Methylation analysis showed that the promoter and the intron 2 of beclin 1 were aberrantly methylated in the tumors with decreased expression.

**Conclusions:**

These data indicated that LOH and aberrant DNA methylation might be the possible reasons of the decreased expression of *beclin 1 *in the breast tumors. The findings here shed some new light on the regulatory mechanisms of beclin 1 in breast cancer.

## Background

Autophagy is a process of cellular protein degradation through the autophagosomic-lysosomal pathway, which plays an important role in cell differentiation and maintenance of cellular homeostasis. However, it is usually defective in tumor cells [1,2]. *Beclin 1*, the mammalian orthologue of the yeast *Atg6/Vps30 *gene, is the first identified tumor suppressor gene in human to mediate autophagy [3,4]. It was originally isolated by a yeast-two-hybrid screen and its protein was identified as an interacting partner of Bcl-2, an important anti-apoptosis protein [5]. Beclin 1 has a regulatory role in the process of vesicle nucleation of autophagy [5,6]. Previous studies demonstrated that over-expression of *beclin 1 *induced apoptosis via activation of caspase-9 in gastric cancer cells [7], while partial silencing of *beclin 1 *aggravated apoptosis in hepatic cancer cells [8]. The different effects of *beclin 1 *on cell death and cell survival in different cells depend on the cellular context.

*Beclin 1 *was mapped to a tumor susceptibility locus approximately 150 kb centromeric to *BRCA1 *on human chromosome 17q21 [9]. Allelic loss of chromosome 17q21 is often found in human prostate, breast and ovarian cancer [10-13]. *Beclin 1 *encodes an evolutionarily conserved 60 kDa coiled coil protein that is widely expressed in human normal adult tissues [9]. It has been reported that reduced levels of *beclin 1 *expression and mono-allelic deletion were observed in human breast cancer cell lines and tissues [9]. Whether there are other mechanisms for the loss of *beclin 1 *expression in breast cancer remains to be determined.

DNA methylation is the major epigenetic modification that involves alterations of chromatin structure. There are increasing evidences that aberrant methylation of CpG islands in 5' regulatory region of tumor suppressor gene leads to transcriptional silencing in cancer [14-16]. The human *beclin 1 *gene contains a 1.5 kb CpG island from the promoter to part of the intron 2, suggesting that DNA methylation may be responsible for down-regulation of *beclin 1 *expression in cancer. In addition, the promoter-associated CpG island of *beclin 1 *contains E2F target site and four putative consensus Sp1 binding sites [17]. In the present study, we detected the mRNA and protein expression levels of beclin 1 and explored the possible effects of DNA methylation and LOH on decreased gene expression in breast cancer tissues. The results here provided some new insights into the regulation of *beclin 1 *in breast cancer.

## Methods

### Tissue samples

20 pairs of tumors and adjacent normal tissues from newly diagnosed patients with sporadic breast invasive ductal cancer (IDCs) were collected from the First Affiliated Hospital of China Medical University after the approval of Institutional Review Board and patients' informed consents. All the patients were females without family hereditary breast cancer. Radio and chemo therapy was not applied to the patients before surgical operation. The patients were consecutive cases. The median age of these patients was 48.5 year-old (range, 40 - 74). The clinicopathologic parameters, including patient's age, tumor size, tumor grade, lymph node status, estrogen receptor (ER), progesterone receptor (PR) and human epidermal growth factor receptor-type 2 (HER2) immunoreactivity were obtained from clinical records. The tissues were obtained after surgical resection and subsequently microdissected with the assistance of pathologists. The corresponding adjacent normal tissues were derived from sites adjacent at least 1 cm away from the tumors. Tissues for immunohistochemistry use were fixed in 10% buffered formalin, embedded in paraffin, and sectioned with a microtome. Sections were stained with hematoxylin and eosin for histological examination by at least two pathologists. Tissue fragments were immediately frozen and stored in liquid nitrogen till used.

### RNA extraction and quantitative reverse transcription PCR

Total RNA from tissues was extracted using TRIZOL reagent (Invitrogen) according to the manufacture's protocol. The first strand cDNA was obtained from total RNA (0.5 μg) and oligo (dT) using the Reverse Transcription System (Promega). For real-time quantitative RT-PCR, gene specific primers and TaqMan fluorescent hybridization probes were used. β-actin was used to normalize the quantity of specific mRNA. The sequences of *beclin1 *and β-actin primers are listed in Table 1. The PCR product was 85 bp for *beclin1 *and 295 bp for β-actin. Their authenticity was confirmed by DNA sequencing. The amplification efficiency determined for both target and housekeeping genes was equal. Relative expression levels were calculated by the 2^-ΔΔCt ^method [18]. Each assay was done in triplicate.

**Table 1:** Nucleotide sequences of primers used

Name	Sequence (5'→3')	Purpose
Bl-F	TGCAACCTTCCACATCT	RT-PCR
Bl-R	TTCCACGGGAACACTG	RT-PCR
β-actin-F	TCACCCACACTGTGCCCATCTACGA	RT-PCR
β-actin-R	CAGCGGAACCGCTCATTGCCAATGG	RT-PCR
Bl-QF	TCTGCCTTCCTCTGTAG	Q-PCR
Bl-QR	TTCCACGGGAACACTG	Q-PCR
18S-QF	ACATCCAAGGAAGGCAGCAG	Q-PCR
18S-QR	TTCGTCACTACCTCCCCGG	Q-PCR
Bl-Taqman	FAM-CACAGTGGACAGTTTGGCACAATCA-TAMRA	Q-PCR
β-actin-Taqman	FAM-CAGCCGTGGCCATCTCTTGCTCGAA-TAMRA	Q-PCR
18S-Taqman	FAM-CGCGCAAATTACCCACTCCCGA-TAMRA	Q-PCR
Bl-bs1F	GTTTTTTAAAGTGTTGGAATTATAAG	Bisulfite first PCR
Bl-bs1R	AACTCCTAATCCACAAACTCACAA	Bisulfite first PCR
Bl-bs1F'	TTGTTGTTGTTTTGAGATGGAGTT	Bisulfite nested PCR
Bl-bs1R'	AAAAATATAAAAACCAAAACC	Bisulfite nested PCR
Bl-bs2F	GGGTTTGTGAGTTTGTGGATTAG	Bisulfite first PCR
Bl-bs2R	AAAAAAAACTCCAATAAAAACC	Bisulfite first PCR
Bl-bs2F'	AGTTTGTGGATTAGGAGTTTTTGTT	Bisulfite nested PCR
Bl-bs2R'	TAAAAATTCCCAAACTCCCTTCTA	Bisulfite nested PCR
Bl-bs3F	ATTTTAGAAGGGAGTTTGGGAATT	Bisulfite first PCR
Bl-bs3R	TTAAACCCTTCCATCCCTAAAAC	Bisulfite first PCR
Bl-bs3F'	TTTTGGGTTTTAAATTGTTTTTGTT	Bisulfite semi-nested PCR
Bl-bs3R'	TTAAACCCTTCCATCCCTAAAAC	Bisulfite semi-nested PCR
Bl-bs4F	ATATTGTGGATTTTTGAGAGTTTTT	Bisulfite first PCR
Bl-bs4R	AAATCTTTCTTTTACTACTAAAAACTCTCT	Bisulfite first PCR
Bl-bs4F'	TTGTAATTTTAGTATTTTGGGAGAT	Bisulfite nested PCR
Bl-bs4R	CTCTATTACCCAAACTAAAATACAATAATA	Bisulfite nested PCR
D17S579F	AGTCCTGTAGACAAAACCTG	Microsatellite analysis
D17S579R	CAGTTTCATACCAAGTTCCT	Microsatellite analysis

Q-PCR, quantitative PCR.

### Genomic DNA extraction and quantitative PCR

Genomic DNA of tumors and matched adjacent normal tissues was extracted with the Dneasy Tissue Kit (Qiagen). A Taqman-based quantitative PCR method using PRISM 7300 Real-Time PCR Thermal Cycler (Applied Biosystem) was applied to detect the relative copy number of beclin 1. DNA content per haploid genome was normalized to that of 18S rDNA and calculated by the 2^-ΔΔCt ^method [18]. Table 1 showed the sequences of the specific primer pairs and the Taqman probes. PCR products were sequenced to confirm their authenticity. Each assay was done in triplicate. A dilution series was performed to quantify primer efficiency.

### Mutational and microsatellite analysis

To search for mutations of the *beclin 1 *gene by polymerase chain reaction - single strand conformation polymorphism (PCR-SSCP) analysis, each of the 12 exons was amplified using its specific primers. The sequences of the primers and PCR conditions were as described by Aita et al [9]. Denatured PCR products were loaded onto non-denaturing 6% polyacrylamide gels, electrophoresed at 4°C, stained with silver staining, and visualized.

To analyze LOH of the *beclin 1 *gene, a microsatellite marker on 17q21, D17S579, was selected through UniSTS database of National Center for Biotechnology Information (NCBI). Primer sequences are shown in Table 1. We defined LOH as a completely absent or significantly decreased signal intensity of one allele.

### Sodium bisulfite modification and sequencing

The methylation status of the *Beclin 1 *CpG island was determined by the bisulfite sequencing method [19]. 0.5~1 μg genomic DNA was digested with BamHI that does not cut within the sequence of the CpG island. DNA was denatured by 0.3 M NaOH at 42°C for 30 min. Then 3 M sodium bisulfite (Sigma) and 10 mmol/L hydroquinone were added, which was further incubated at 50°C for 16 h. Modified DNA was purified using Wizard DNA purification resin (Promega), re-treated with 0.3 M NaOH at 37°C for 15 min, precipitated with ethanol, and resuspended in 50 μL water. Semi-nested or nested PCR was used to amplify these regions. Primer sequences are detailed in Table 1. Five clones of each PCR sample were picked up and sequenced.

### Immunohistochemistry

Protein expression of *beclin 1 *was performed on breast tissues fixed by formalin and embedded by paraffin. Slides of 4 μm sections were deparaffinized with xylene and antigen retrieval was accomplished by heat. The sections were then incubated in 3% hydrogen peroxide at room temperature for 5 min to block endogenous peroxidase activity. Slides were then incubated with rabbit anti-beclin 1 polyclonal antibody (Cell Signaling) at 1:300 dilutions at 4°C overnight. Then rinse the slides three times in PBS for 5 m each and incubate in biotin-labeled rabbit anti-rabbit secondary antibodies for 1 h at room temperature. After washing three times with PBS, the staining was performed using 3, 3'-diaminobenzidine. Sections were counterstained with hematoxylin. Staining was considered positive when cytoplasmic staining was observed in at least 30% of the neoplastic cells. And staining intensity was the strength of the signal and was evaluated on a scale of 0 to 2: 0, negative; 1+, weak staining; 2+, moderate/strong staining. We also compared IHC data between tumors and corresponding adjacent normal tissues by percentage of positive cells and intensity of staining to estimate the changes of beclin 1 expression.

P53 protein expression was performed using anti-p53 monoclonal antibody (Ab-8, Lab Vision Corporation), which recognized both wild type and mutant forms of the p53 protein. BRCA1 and BRCA2 protein expression were assessed using an anti-BRCA1 monoclonal antibody (Ab-1, Oncogene Research Products) and an anti-BRCA2 polyclonal antibody (Ab-2, Oncogene Research Products). Samples were considered positive when 20%, 10%, and 10% of the cells were stained with p53, BRCA1 and BRCA2, respectively.

### Data analysis

Data were expressed as mean ± standard deviation. The ratio of *beclin 1 *mRNA expression in the tumor to that in the corresponding adjacent normal tissue in each case was transformed using a common logarithm. For statistical comparisons of these log-transformed data between two groups, a t-test was used. All statistical analyses were performed using SPSS software, version 10.0 (SPSS Japan Inc., Tokyo, Japan). For all of the statistical tests, a two-sided p-value of less than 0.05 was considered statistically significant.

## Results

### Down-regulated beclin 1 mRNA expression in breast cancer tissues

**Figure 1:**
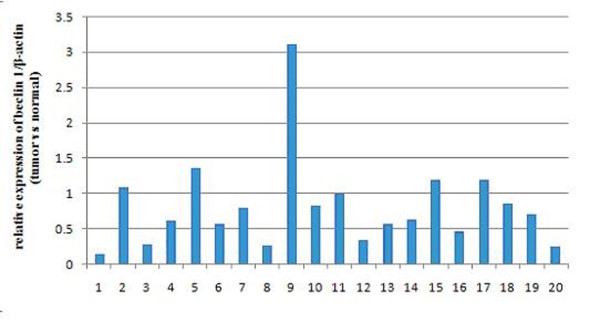
***Beclin 1 *mRNA relative expression of breast tumors vs adjacent normal tissues**. Quantitative RT-PCR was carried out to detect the expression of beclin 1 with β-actin as an normalizer.

We detected relative mRNA expression of *beclin 1 *of 20 breast tumor tissues vs. corresponding normal tissues by quantitative RT-PCR. The mean ± SD *beclin 1/*β-actin mRNA level was 1.56 ± 1.01 for tumor tissues and 2.24 ± 1.39 for adjacent normal tissues (Table 2). As a whole, down-regulated expression of *beclin 1 *was found in 14 out of 20 (70%) of the breast cancer tissues (Figure 1). Furthermore, 6 out of 20 (30%) in the breast tumors showed a significant decrease (tumor: normal tissue ratio < 0.5) of *beclin 1 *mRNA expression. Meanwhile, down-regulated *beclin 1 *mRNA expression was not observed in 6 out of 20 (30%) of the breast tumors compared to the corresponding normal tissues.

**Table 2:** *Beclin 1*/β-actin mRNA expression in breast tissues

n = 20	Beclin 1/β-actin mRNA expression
Adjacent normal tissues	2.24 ± 1.39
Tumors	1.56 ± 1.01
	*p *= 0.034

Data are means ± SD. The numbers show the mean values of *beclin 1*/β-actin mRNA expression and standard deviation in 20 breast tumors and adjacent normal tissues. Statistical analysis was done by paired *t *-- test.

As was shown in Table 3, we analyzed the correlation between *beclin 1 *mRNA expression and clinicopathological parameters, including age, the presence of lymphatic invasion, estrogen receptor (ER), progesterone receptor (PR) and human epidermal growth factor receptor-type 2 (HER2). No detectable significant difference was observed.

**Table 3:** Correlation between *beclin 1 *mRNA expression in breast cancer and clinicopathologic parameters

	Case number	Average of log T/N	Standard deviation of log T/N	P-value
Age (y)				
50	11	-0.13	0.06	0.160
≥50	9	-0.30	0.07	
Lymphatic invasion				
Positive	8	-0.23	0.06	0.356
Negative	11	-0.13	0.05	
ER				
Positive	7	-0.31	0.08	0.199
Negative	13	-0.15	0.06	
PR				
Positive	10	-0.20	0.06	0.905
Negative	10	-0.21	0.09	
HER2				
Positive	19	-0.21	0.07	-
Negative	1	-0.07	-	

T, tumors; N, normal tissues; -, not applied.

### Immunohistochemical analysis of beclin 1 protein expression in breast tumors

**Figure 2:**
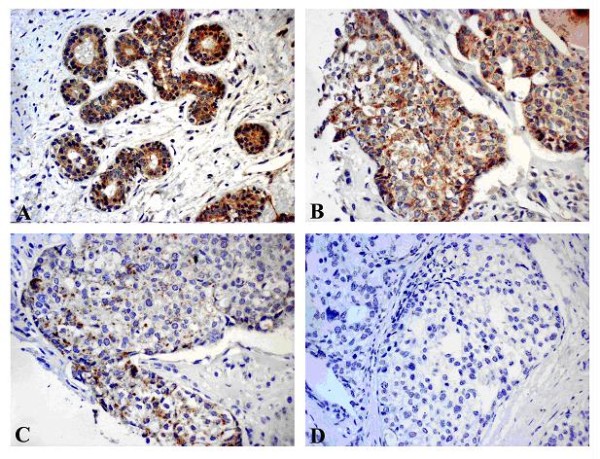
**Immunohistochemical analysis of beclin 1 expression in breast tissues**. Photographs were taken at × 200 magnification. A brown color represents positive staining of beclin 1. Counterstaining of nuclei was performed with hematoxylin as a blue color. (A) Positive staining of beclin 1 in adjacent normal breast epithelial cells; (B) Positive staining of beclin 1 in a breast tumor; (C) Weak immunoreactivity of beclin 1 in a breast tumor; (D) Negative control of beclin 1 staining in a breast tumor.

Sections of breast tumors were applied to analyze the protein expression of beclin 1. The staining was cytoplasmic. 13 out of 20 (65%) of tumors showed reduced beclin 1 staining (Figure 2C, 2D) and normal epithelial cells showed strong cytoplasmic beclin 1 expression (Figure 2A). As illustrated in Table 4, there was significant positive correlation between *beclin 1 *mRNA expression and immunoreactivity in breast tumors (p < 0.001).

**Table 4:** Comparison between *beclin 1 *mRNA and protein expression in breast cancer

Beclin 1 mRNA expression	Beclin 1 protein expression (IHC)	
**(Quantitative RT-PCR)**	**Positive**	**Negative**

Positive (6)	6	0
Negative (14)	1	13
	*p *< 0.001	

Positive means that the expression level of beclin 1 mRNA/protein in breast tumors increased compared with the corresponding adjacent normal tissues. And negative means that the expression level of beclin 1 mRNA/protein in breast tumors decreased compared with the corresponding adjacent normal tissues. The association between beclin 1 mRNA and protein expression was analyzed using the χ^2 ^test.

### The correlation between beclin 1 mRNA expression and p53, BRCA1 and BRCA2 protein expression in breast tumors

**Figure 3:**
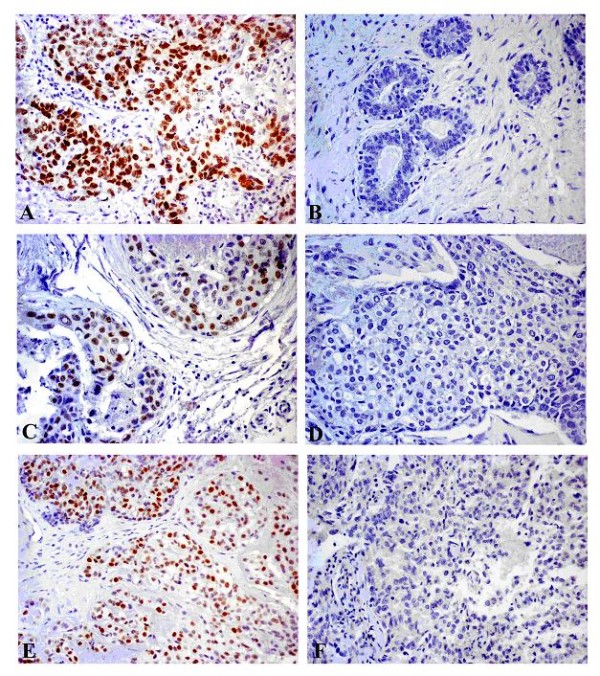
**P53, BRCA1 and BRCA2 staining in breast tumors**. Signals were photographed in breast tissues at × 200. (A) Positive nuclear staining of p53 protein; (B) No obvious nuclear staining was observed over the whole normal breast tissue; (C) Positive nuclear staining of BRCA1 protein; (D) Negative control of BRCA1 staining in a breast tumor. (E) Positive nuclear staining of BRCA2 protein; (F) Negative control of BRCA2 staining in a breast tumor.

P53 protein over-expression was detected in 2 (10%) out of 20 breast tumors (Figure 3A, Table 5). There was no obvious excess nuclear p53 protein staining in the corresponding normal tissues, which indicates that the p53 level in the normal tissues is low (Figure 3B). BRCA1 and BRCA2 positive nuclear staining was observed in 6 (30%) and 9 (45%) out of 20 breast tumors, respectively (Figure 3B, 3D, Table 5). *Beclin 1 *mRNA levels were significantly higher in the BRCA1 positive tumors than those in the BRCA1 negative ones (p = 0.014) (Table 6).

**Table 5:** Beclin 1 mRNA and protein expression, p53, BRCA1 and BRCA2 protein expression and LOH analysis of *beclin 1 *in breast cancer tissues

Breast cancer cases	Beclin 1 mRNA expression	Protein expression				LOH (Q-PCR)	LOH (microsatellite)
				
		Beclin 1	P53	BRCA1	BRCA2		
1	-	-	-	-	-	+	+
2	+	+	-	+	+	-	-
3	-	-	-	-	-	+	+
4	-	-	-	-	+	+	-+
5	+	+	-	+	-	-	-
6	-	-	-	+	+	+	+
7	-	-	-	+	+	+	+
8	-	-	-	-	-	+	+
9	+	+	-	-	-	-	-
10	-	-	-	+	-	-	-
11	+	+	+	-	+	+	+
12	-	-	-	-	-	+	+
13	-	-	-	-	+	+	+
14	-	-	-	-	-	+	+
15	+	+	-	-	-	-	-
16	-	-	-	-	-	-	-
17	+	+	+	+	+	+	-
18	-	+	-	-	-	-	-
19	-	-	-	-	+	-	-
20	-	-	-	-	+	-	-

"+": positive; "-": negative.

**Table 6:** Relationship between *beclin 1 *mRNA expression and immunohistochemical characterization of p53, BRCA1 and BRCA2 in breast IDCs

	Case number	Average of log	Standard deviation	P-value
	**(%)**	**T/N**	**of log T/N**	

*p*53				
Positive	2 (10)	-0.24	0.24	0.798
Negative	18(90)	-0.20	0.06	
BRCA1				
Positive	6 (30)	-0.01	0.02	0.014*
Negative	14 (30)	-0.31	0.08	
BRCA2				
Positive	9 (45)	-0.16	0.04	0.483
Negative	11 (55)	-0.24	0.10	

No obvious nuclear p53 protein staining was defined as negative and over-expression of p53 was defined as positive. Loss or reduction of BRCA1 and BRCA2 expression was defined as negative in tumors compared with the paired normal tissue, and vice versa.

### LOH of beclin 1 was detected in some of the breast cancer tissues

**Figure 4:**
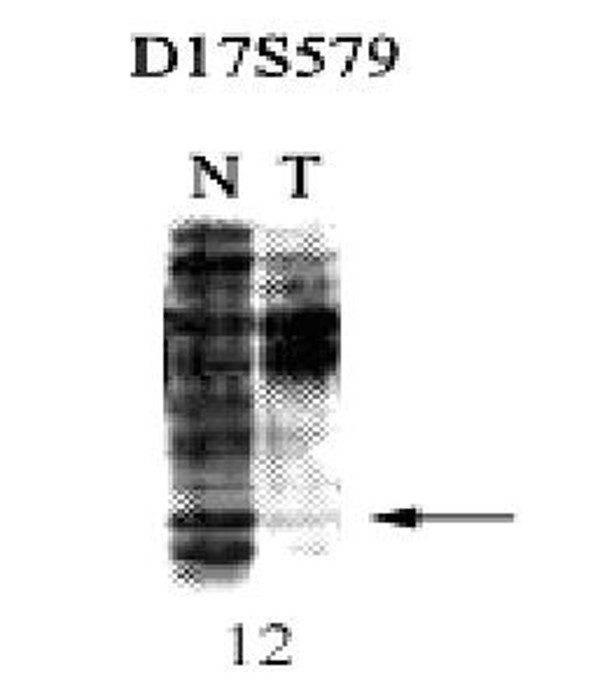
**Representative photograph showing LOH in breast cancer**. Above graph, microsatellite marker; under graph, patient's number; arrowheads, the allele losses; N, normal DNA; T, tumor DNA.

Using quantitative PCR method, *beclin 1 *gene copy number was detected in all breast tissues. As was shown in Table 5, LOH was found in 11 out of 20 (55%) of breast tumors (tumor/normal < 1.0). Meanwhile, we applied a microsatellite marker, D17S579 [10], to validate the sensitivity of this methodology. LOH was verified in 9 out of 20 (45%) of breast tumors (Figure 4, Table 5). Table 7 compared the correlation between both methods for LOH analysis. The results showed a significant relationship (p < 0.001) between both methods, which exhibited accordance in 90% of cases.

**Table 7:** Comparison between microsatellite and quantitative -- PCR method for LOH analysis of the *beclin 1 *gene in 20 paired tissues

Q-PCR	D17S579	
	**Negative**	**Positive**

Positive (13)	2	11
Negative (7)	7	0
	*p *< 0.001	

D17S579 was a microsatellite marker located in 17q21. Comparison was analyzed using the χ^2 ^test.

### CpG island analysis in beclin 1 regulatory region

**Figure 5:**
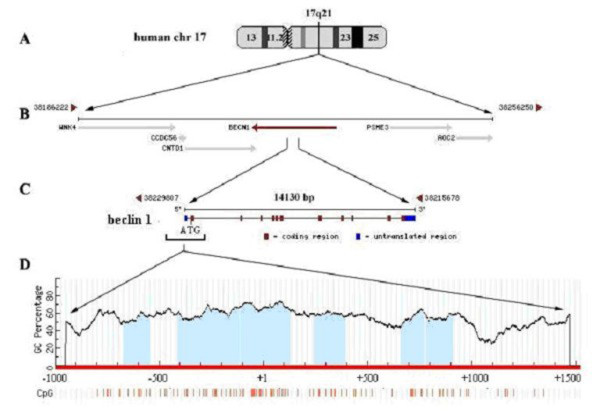
**Genomic architecture of the human *beclin 1 *gene**. The genome database of National Center for Biotechnology Information (NCBI) and MethPrimer program were used to inspect the *beclin 1 *locus and CpG island, respectively. (A) Location of the *beclin 1 *gene within human chromosome 17. (B) 70-kb region flanking the *beclin 1 *gene and its relative location with neighbor genes. (C) Exon/intron structure of the human *beclin 1 *gene. (D) Graph of percent guanine (G) and cytosine (C) nucleotides across 5' region of the *beclin 1 *gene and location of CpG dinucleotides.

Previous studies reported that *beclin *1 maps to 17q21 that spans over 12 kb locus of human genome (Figure 5A) [9]. The 70 kb region of genome in which the *beclin 1 *gene residues, contains a moderate number of genes (Figure 5B). Telomerically flanking the *beclin 1 *gene is the *PSME3 *gene, a member of the *PA28 *family, which binds specifically to 20 S proteosomes and stimulates the hydrolysis of peptides [20], and the *AOC2 *gene, a member of the copper-binding amine oxidase super family, which is an important regulator of cellular polyamine levels [21]. Centromerically flanking *beclin 1 *is the WNK4 gene, known to regulate thiazide-sensitive Na-Cl co-transport [22], and the other two genes, CNTD1 and CCDC56 with unknown functions. *Beclin *gene is composed of 12 exons and exon 2 encodes the translational initial codon (ATG) (Figure 5C). 5' region of the *beclin 1 *gene was inspected by the MethPrimer program http://www.urogene.org/methprimer. The data showed this region of the genome to be a CG rich (Figure 5D). The CpG island encompassed from the promoter to the intron 2 (nt -528 to 977). Because aberrant cytosine methylation within 5' end of tumor suppressor genes is commonly observed in cancer cells [23,24], *beclin 1 *was likely a candidate gene for epigenetic silencing.

### Certain CpG dinucleotides in the promoter and the intron 2 of the beclin 1 gene were methylated in breast tumors

**Figure 6:**
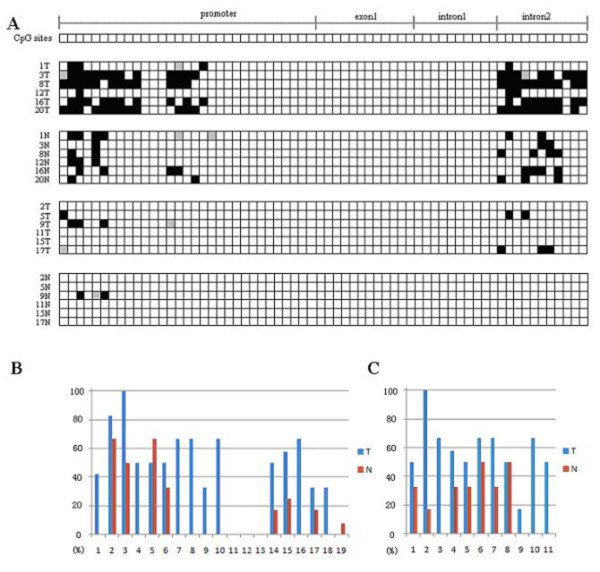
**Methylation status of *beclin 1 *gene in different cases**. (A) Methylation patterns of the *beclin 1 *gene in breast tumors and adjacent normal tissues. Sequencing of bisulfite treated DNA revealed methylation patterns for *beclin 1 *gene. Black squares represent methylated CpG dinucleotides, white squares represent unmethylated CpG dinucleotides and gray squares are hemimethylated. Hemimethylated (grey squares) means that only one C allele in the same CpG site of the two DNA strands is methylated. Methylated (dark squares) means that both C alleles in the same CpG site of the two DNA strands are methylated and unmethylated means that both C alleles in the same CpG site of the two DNA strands are unmethylated. 1, 3, 8, 12, 16 and 20: samples with significant decreased expression of *beclin 1*; 2, 5, 9, 11, 15 and 17: samples without decreased expression of beclin 1 in tumors. (B) Percentage of methylation status from 1^st ^to 19^th ^CpG site in the promoter of *beclin 1 *in 6 cases with significant decreased mRNA expression. (C) Percentage of methylation status from 1^st ^to 11^th ^CpG site in intron 2 of *beclin 1 *in 6 cases with significant decreased mRNA expression.

There are genetic and epigenetic alterations for the down-regulation of many genes. To identify mutations in the *beclin 1 *gene in breast cancer, mutational anlaysis was carried out by PCR-SSCP in genomic DNA of 20 breast tumors. No mutation was found in 12 exons of the beclin 1 gene in 20 breast tumors. As mentioned above, there was a large and dense CpG island ranging from the promoter to the intron 2 of the *beclin 1 *gene. Therefore, we first detected the methylation status of the CpG islands in the 6 breast tumors with significantly down-regulated beclin 1 expression and corresponding normal tissues by bisulfite sequencing. In the promoter from -528 to -65 (from transcription start site) and the intron 2 from 733 to 977, methylation was detected in 4 tumors (T3, 8, 16, 20) and weakly detected in the corresponding normal tissues. In the other two samples (T1, 12) with significantly down-regulated *beclin 1 *expression and LOH, methylation was weakly detected in both tumors and corresponding normal tissues. Then we tested another 6 cases, whose *beclin 1 *mRNA expression was not down-regulated in breast tumors, and methylation was weakly or hardly detected in both tumors and normal tissues (Figure 6).

## Discussion

It has been reported that tumor suppressor genes with high frequencies of LOH in human chromosomal region 17q21 and epigenetic silencing, are important in hereditary and sporadic breast cancer tumorigenesis [25,26]. One of these genes is *BRCA1*, whose decreased expression was often observed in breast cancer with epigenetic silencing and mono-allelic deletion of the *BRCA1 *gene [27-29]. In the present study, we showed that the mRNA and protein expression of *beclin 1*, another 17q21 gene, was frequently down-regulated in breast tumors. This down-regulation in some tumors was due to allele loss in gene copy number and in some others was due to DNA methylation. There was no significant association between *beclin 1 *mRNA expression and clinocopathologic parameters.

P53, BRCA1 and BRCA2 are clearly involved in the development of both sporadic and hereditary breast cancers [30-34]. Mutations of these genes are the most common genetic variations and are widely distributed in breast cancer cells. Tumors with missense mutations displayed positive nuclear immunoreactivity of p53 for the effect of mutation on the prolongation of p53 half-life through increased protein stability [35]. Cancers with wild-type P53, including breast cancer [35], are generally negative for p53 immunoreactivity due to the rapid degradation of normal p53 protein. Meanwhile, loss or reduction of BRCA1 and BRCA2 expression has been exhibited in sporadic breast cancers [36]. Immunohistochemistry of p53 and BRCA1/2 proteins in tumor cells may be useful as an additional method in exploring the relationship between the beclin 1 expression levels and the status of these important tumor suppressor genes in breast cancer. Therefore, we examined their expression levels by immunohistochemcial staining in 20 breast tumors. By IHC staining of p53 protein, we found 2 cases (T11 and T17) exhibited more than 50% positive nuclear staining cells of all tumor cells. IHC over-expression of p53 might reflect that genetic alterations also occurred in these tumors. We further analyzed the correlation between *beclin 1 *expression and p53 over-expression and no obvious association was obtained. It is known that the normal BRCA1 protein plays an important role in repairing breaks in DNA [37]. However, when *BRCA1 *exhibited loss of expression due to genetic or epigenetic alterations, abnormal repair function of BRCA1 may lead to DNA replication errors and cancerous growth [38,39]. Liang *et al *[3] reported that *beclin 1 *played a negative regulatory role in mammary cell growth and tumorigenesis using gene-transfer techniques. Our data showed that *beclin 1 *expression was significant higher in the BRCA1 positive tumors than in the negative ones, suggesting *beclin 1 *expression may be related to cell growth in breast cancer.

*Beclin 1 *is mapped to a region approximately 150 kb to *BRCA1 *on chromosome 17q21, which is usually deleted in breast, ovarian and prostate cancer [8-11]. LOH at the *beclin 1 *locus was observed in 9 out 22 (41%) of the breast cancer cell lines [9]. In the present study, all examined tumors exhibited a LOH rate of at least 45% at the *beclin 1 *locus and no mutation was found in the coding regions of *beclin 1*. These results confirmed that frequent allelic losses of *beclin 1 *were part of reasons for *beclin 1 *down-regulation and mutations were unlikely the main regulatory mechanism for *beclin 1 *inactivation in breast cancer. Meanwhile, allelic loss of *beclin 1 *was only found in a portion of the breast tumors with down-regulated expression, indicating that mechanisms other than allelic deletion may be responsible for the decrased mRNA expression.

The CpG island in *beclin 1 *spans over 1.5 kb from the promoter to the intron 2 [nucleotide (nt) -528 to 977] with the transcription start site defined as +1, so it is divided into four regions to be cloned. The first region contained part of the beclin 1 promoter from -528 to -65. The second region was located between -83 and 164 from the proximal promoter to part of the first intron. The third region laid between 137 and 400 from a portion of the first intron to part of the second exon. The fourth region was situated between 733 and 977 from a portion of the second intron. We found that certain CpG dinucleotides at the promoter and the intron 2 of the *beclin 1 *gene are hypermethylated in breast tumors. As dense methylation of the 5' CpG islands was not detected in normal tissues, we speculated that this methylation patterns in tumor cells were aberrant and disease associated. As a whole, the regulation of *beclin 1 *expression was a little complicated. In some cases, T16 and T20, the decreased expression was due to aberrant DNA methylation; While in T1 and T12, the decrease was from LOH; And in the other cases, T3 and T8, both aberrant DNA methylation and LOH devoted to the decreased expression of *beclin 1 *(Table 8). For this reason, different mechanisms seemed to be involved in the regulation of *beclin 1 *expression.

**Table 8:** Summary of LOH and aberrant DNA methylation in breast tumors showing down -- regulated expression of *beclin 1*

**No**.	LOH	DNA Methylation
1	+	-
3	+	+
8	+	+
12	+	-
16	-	+
20	-	+

Some tumors (T10 and T19) with low expression of *beclin 1 *did not have either LOH or methylation. Other identified mechanisms might contribute to the regulation of beclin 1 expression, such as bcl-2 overexpression [40]. Recently, a novel beclin 1 binding protein, UVRAG, was found to positively regulate autophagy signaling pathway mediated by beclin 1 in colon cancer [41]. Exploring different involving factors that could affect the regulatory expression of *beclin 1 *might contribute to the pathogenesis of human cancers.

Aberrant methylation of 5' CpG islands associated with down-regulated mRNA expression of *beclin 1 *indicated that methylation might be a new mechanism for loss of expression of *beclin 1*, which has not been shown previously. Sequence analysis revealed that there were four putative consensus Sp1 binding sites at the promoter [17] and one putative Sp1 binding site at the intron 2, which share high sequence homology to the consensus Sp1 motif. It has been reported that aberrant DNA hypermethylation in the promoter of a gene can silence its expression [42], and that methylation in the introns can silence or enhance its expression [43-45]. Whether DNA methylation in the promoter and the intron 2 of *beclin 1 *affects the binding affinity of transcriptional factor needs to be further determined.

## Conclusions

In conclusion, our studies confirmed that down-regulation of *beclin 1 *expression is present in breast cancer. Other than deletion in gene copy number, DNA hypermethylation in the promoter and/or intron 2 may be a new mechanism responsible for down-regulation of *beclin 1 *expression. Since *beclin 1 *has important functions in apoptosis and autophagy, its epigenetic modification might provide new targets for cancer therapy.

## Competing interests

The authors declare that they have no competing interests.

## Authors' contributions

LZD carried out molecular biological studies, participated data collection, analysis and the preparation of the manuscript. CB and JF collected tissue samples and participated in the clinical part of the study and immunohistochemistry. WYQ, XYJ and LXJ supervised the project and overviewed the analysis of the data and the manuscript. All authors have read and approved the present manuscript.

## Pre-publication history

The pre-publication history for this paper can be accessed here:

http://www.biomedcentral.com/1471-2407/10/98/prepub
